# Susceptibility of bovine dental enamel with initial erosion lesion to new erosive challenges

**DOI:** 10.1371/journal.pone.0182347

**Published:** 2017-08-17

**Authors:** Gabriela Cristina de Oliveira, Guida Paola Genovez Tereza, Ana Paula Boteon, Brunna Mota Ferrairo, Priscilla Santana Pinto Gonçalves, Thiago Cruvinel da Silva, Heitor Marques Honório, Daniela Rios

**Affiliations:** 1 Department of Pediatric Dentistry, Orthodontics and Public Health Bauru School of Dentistry, University of São Paulo, Bauru, Brazil; 2 Department of Operative Dentistry, Endodontics and Dental Materials Bauru School of Dentistry, University of São Paulo, Bauru, Brazil; 3 Department of Prosthodontics Bauru School of Dentistry, University of São Paulo, Bauru, Brazil; University of Florida, UNITED STATES

## Abstract

This *in vitro* study evaluated the impact of initial erosion on the susceptibility of enamel to further erosive challenge. Thirty bovine enamel blocks were selected by surface hardness and randomized into two groups (n = 15): GC- group composed by enamel blocks without erosion lesion and GT- group composed by enamel blocks with initial erosion lesion. The baseline profile of each block was determined using the profilometer. The initial erosion was produced by immersing the blocks into HCl 0.01 M, pH 2.3 for 30 seconds, under stirring. The erosive cycling consisted of blocks immersion in hydrochloric acid (0.01 M, pH 2.3) for 2 minutes, followed by immersion in artificial saliva for 120 minutes. This procedure was repeated 4 times a day for 5 days, and the blocks were kept in artificial saliva overnight. After erosive cycling, final profile measurement was performed. Profilometry measured the enamel loss by the superposition of initial and final profiles. Data were analyzed by t-test (*p*<0.05). The result showed no statistically significant difference between groups (GS = 14.60±2.86 and GE = .14.69±2.21 μm). The presence of initial erosion on bovine dental enamel does not enhance its susceptibility to new erosive challenges.

## Introduction

Dental erosion is defined as the tooth surface loss caused by acids of non-bacterial origin[1–3] The pathophysiology of dental erosion, however, is more complex than previously described[4]. Erosion is currently considered a near surface demineralization, comprising two developmental stages. The initial erosive stage, termed dental erosion, corresponds to enamel softening, which results in losses of mechanical resistance and structural integrity [5, 6]. The following process, so-called erosive tooth wear, occurs by either prolonged demineralization of tooth surface or the action of mechanical forces onto the softened area, leading to irreversible dental enamel loss[5–7].

Because the prevalence of erosion is high[8–10], studies have been conducted to search preventive methods and early treatments for erosive tooth loss[11–13]. Many of them were conducted *in vitro* and despite their limitations, these studies have been used as an important tool in testing the effectiveness of methods to prevent erosion, since clinical trials present low accuracy of available methods for the measurement of tooth tissue loss[14]. Laboratory protocols for dental erosion are diverse in type, exposure periods to the acids[15–18], types of saliva[19] and developmental stage of enamel erosive lesion[18, 20–24]. Regarding the stage of the erosive lesion, studies have been conducted on noneroded[18, 22, 23] or eroded enamel[20, 21, 24]. Generally, noneroded enamel is used to test a given treatment to prevent the occurrence of erosion by simulating a healthy patient at risk in developing dental erosion. On the other hand, eroded enamel is employed to test treatments capable of inhibiting the progression of erosion, attempting to mimic a patient already with the disease[25, 26]. Since the eroded softened layer might not be totally rehardened by saliva[19, 27–29] this layer could influence the susceptibility to erosion. However, to the best of our knowledge, the impact of an erosive challenge on eroded enamel has not been investigated yet.

Therefore, this study aimed to evaluate the susceptibility of the tooth enamel with initial erosion lesion to new erosive challenges in relation to noneroded enamel.

## Material and methods

This study was conducted *in vitro* and the factor under study was enamel susceptibility to erosive tooth wear at two levels, noneroded enamel (GC-control) and enamel with initial erosion lesion (GT-test). Thirty bovine enamel specimens were randomly divided into two groups. The sample of 15 specimens for each group was calculated based on a pilot study, considering an estimated standard deviation of 1.39 μm, an expected difference in means of 1.5 μm, an alpha error of 5% and a beta error of 20%. The initial erosion lesion of group GT was produced *in vitro* through the immersion of the specimens into HCl 0.01 M, pH 2.3 for 30 s. Next, all specimens were subjected to erosive cycling for 5 days. The null hypothesis was that there would not be difference in the behavior of noneroded and previously eroded dental enamel against erosive challenge. To test this hypothesis, the response variable used was the enamel loss (μm) analyzed by profilometry.

The enamel specimens were prepared from bovine teeth, which were obtained from the Mondelli Food Industry S.A. (Bauru, São Paulo, Brazil). The crowns were separated from their roots and embedded into self-curing acrylic resin (JET, Campo Limpo Paulista, SP, BR). Following, the embedded specimens were flattened and polished under constant tap water cooling using a Metallographic Polishing Machine (APL 4, Arotec, Cotia) with silicon carbide paper discs (300, 600, and 1200 grade papers; Extec Corp, Enfield, USA), ending with felt paper moistened with 1 μm diamond suspension (Buehler, Ltd., Lake Bluff, IL,USA).

The superficial hardness was evaluated with the aid of a hardness tester (HMV-2000/ Shimadzu Corporation) linked to a computer and specific software to analyze the images (Cams-Win-New Age Industries). The specimens with mean hardness values 10% above or below the general mean were discarded (mean surface hardness of 351 ± 15 KHN). Forty-five specimens were selected, fifteen for group GC and thirty for group GT. Considering possible losses after erosive demineralization, group GT received more specimens.

Initial erosion lesion was obtained *in vitro* by immersing GT specimens into hydrochloridric acid (0.01 M, pH 2.3, 17.6 mL/ specimens) for 30 s, under stirring at 50 rpm speed, and environmental temperature of 25°C[21]. In a pilot study[22], the aforementioned protocol provoked erosion with surface softening but without detectable wear. Superficial hardness was evaluated again to confirm the lesion formation and to select only 15 specimens (mean surface hardness of 171 ± 12 KHN).

The specimens were subjected to erosive cycling for 5 days. Erosive cycling was performed by immersing the specimens into hydrochloridric acid (0.01 M, pH 2.3, 17.6 mL/ specimens) for 2 min. Following the erosive attack, the specimens were washed under deionized water for approximately 20 s, and immersed into artificial saliva[19] for 2 h. This procedure was repeated 4 times per day. At the end of each cycling day, the specimens were immersed in artificial saliva overnight (14 h) at 25°C.

The wear measurement was performed with the aid of a surface profilometer (Mahr Perthometer,Göttingen, Germany), linked to a computer with contour software (MarSurf XCR 20). Before the erosive challenge enamel specimens were marked with a scalpel blade No. 11 (Embramac, Itapira, SP, Brazil) to delimit three areas: two lateral ones (reference areas) and the central area (test area of 2.0 mm2). Specimens were fixed to a device to standardize their position and to allow the record of the location of each profile. Prior to erosive cycling, five readings were executed at determined distances (2.25, 2.0, 1.75, 1.5 and 1.25 μm). The graphics of each read were saved individually. To maintain the integrity of the reference areas during the erosive cycling, the two-thirds located at the borders of the specimens were protected with nail varnish (Maybelline Colorama, Cosbra Cosmetics Ltda, São Paulo, SP, Brazil).

After cycling, the nail varnish was removed from the specimens and new five readings were carried out exactly on the same sites of the initial readings. Following, the initial and final graphs of each five readings were superimposed. The average points were selected for measuring the distance between the graphs in height, defining the enamel loss in millimeters and converted to micrometers.

Data were statistically analyzed by SigmaPlot software for Windows version 12.3 (2011 Systat Software, Germany). Since the assumptions of equality of variances and normal distribution were satisfied T-test was applied. The significant limit was set at 5%. The raw data and the statistical analysis are in S1 Table.

## Results

The results showed that after erosive cycling, both the noneroded enamel and the enamel with initial erosion lesion exhibited similar levels of erosive wear (p>0.05) (Table 1).

**Table 1:** Mean enamel loss and standard deviation of two groups (noneroded and eroded enamel) after the erosive cycling.

Enamel	Enamel loss (μm)*
**Noneroded**	14.60 (±2.86)
**Initial Erosion Lesion**	14.69 (±2.21)

*There was no significant difference between groups. T-test (p>0.05).

## Discussion

Although studies use noneroded or eroded/demineralized enamel as if they present different behaviors, the findings of the present study demonstrated that there were no statistically significant differences in enamel loss between noneorded enamel and enamel with initial erosion lesion for bovine teeth. Two factors that could influence these results should be considered: the intensity of initial erosion lesion and the aggressiveness of the erosive challenge.

The progressive stages of dental erosion includes the softening of the outermost enamel surface by the acid attack, which forms small pores on the enamel surface and demineralization of the near-surface layer[4, 30] that characterizes the initial erosion lesion known as dental erosion[4]. The continuity of acid attacks or the incidence of abrasive forces such as chewing food or toothbrushing result in enamel dissolution in which surface loss occurs, this stage is named erosive tooth wear[4]. This study considered the initial lesion to show surface softening but no erosive wear of the enamel. Far as we are aware, the literature does not report a standardized method to obtain this lesion[15, 26, 29, 31–33].

Rakhmatullina et al.[29] measured the height loss of the indentations on the enamel surface after progressive erosive challenges to evaluate the limit period that the surface was demineralized without losing height. Until 2 min of enamel immersion into citric acid 0.034 M, pH 3.6, no alteration in the height of indentations was observed; however, after 4 minutes, loss was seen. To conduct the present methodology, a pilot study was carried out by sequentially immersing (at 15 s interval) the enamel into HCl 0.01 M, pH 2.3 and verifying the sharpness of the margins of the initial indentations obtained with the aid of Knoop indenter when the enamel was sound[22]. Although this method could be considered less accurate, it produced an initial lesion with a shorter time (30 s). The different acid types, with different pH values, explained and justified the time period differences to obtain lesions with similar features. Consensus exists on the fact that the enamel erosion level caused by erosive beverages, acids, juices and foods is associated with the amount of titratable acid, exposure time, temperature, solution concentration and pH, under different conditions[26, 33–37]. Additionally, in shorter challenges (1–5 min), the erosive capacity is determined mainly by acid pH and type, but not by the amount of titratable acid or acid concentration[33, 38].

In another study, Brevik et al.[39], analysed the enamel hardness after erosive challenge with citric acid 0.034 M, pH 3.6 at 4 min intervals for 32 min and showed the loss of enamel structure at the fourth minute, confirming the previous results of the same group. Additionally, the authors observed a fast hardness loss at the first minutes of erosion, followed by a stabilization period in which hardness remained constant. The authors justified that in the process of the initial lesion, corresponding to the phase of enamel softening, the hardness decreased because of the loss of tooth minerals. The subsequent prolonged challenge results in substance loss, with the remaining superficial enamel becoming softened[39]. By continuing with the erosive challenge, this softened layer reaches balance and does not progress, but enamel structure is still lost(30). The results of Brevik et al.[39] showed that when there is hardness stability, enamel erosive wear probably already occurred. The severe intensity of the erosive challenge to form the initial erosion lesion probably reached this balanced stage of lesion softening with enamel loss in many studies[15, 20, 38]. In this context, it will be of interest also to compare the behavior of the sound enamel with the eroded enamel on a stage of balanced superficial hardness.

Another factor to be discussed is the strength of the erosive challenge. Before performing this study it was hypothesized that a porous surface (initial erosion lesion) would exhibit a greater exposed damaged area which might increase enamel susceptibility to erosive dental wear. However, the present erosive protocol was strong enough to similarly promote surface loss on eroded and noneroded surface. This study employed an erosive challenge very common in the literature[35, 40, 41]. Less aggressive challenges when compared to the one applied in the present study, by either acid type, pH or cycling period could result in a smaller impact on both noneroded and previously eroded enamel. Maybe the initial differences existing between them might be maintained until the end of a less aggressive challenge. On future studies, it is important to evaluate the behavior of noneroded and initially eroded enamel under different degrees of challenge aggressiveness.

It is important to bear in mind that this study used bovine enamel, which is widely used in studies to analyze the effect of various conditions on enamel erosion with good reproducibility of results when comparing to human teeth[18]. In addition, the enamel surface of the specimens was ground and polished, removing the outermost enamel layer. This procedure reduces the variation among the specimens[42]. On the other hand, this outermost layer is hypermineralised by fluoride and saliva during the de- and remineralization processes[43]. Since in the present study this outer enamel was removed by the grinding procedures, it is assumed that the susceptibility to dissolution have been higher when compared to native enamel. However, the interference on results might be minimal because both groups under study were tested on the same condition. In further studies, the impact of initial erosion on the susceptibility of enamel to erosive challenges might also be evaluated *in situ* with human teeth, to better reproduce the clinical situation. When considering in situ models the main advantage is the exposition of the enamel specimens to the oral cavity allowing the contact with saliva, which is the most important biological factor on the etiology of erosion[14].

In conclusion, considering the limitations and the results of this *in vitro* study, the softened enamel resulting from initial erosion is as susceptible to a new erosive challenge as the sound one.

## Supporting information

S1 Table Raw data of enamel loss of the studied groups (noneroded and eroded enamel) and statistical analysis. (XLSX)
